# Impact of Obesity and Hyperglycemia on Pregnancy-specific Urinary Incontinence

**DOI:** 10.1055/s-0043-1770087

**Published:** 2023-07-21

**Authors:** Giovana Vesentini, Fernanda Piculo, Gabriela Marini, Angélica Mércia Pascon Barbosa, José Eduardo Corrente, Marilza Vieira Cunha Rudge

**Affiliations:** 1 Perinatal Diabetes Research Center, Botucatu Medical School, Universidade Estadual Paulista, Botucatu, SP, Brazil; 2 Department of Health Sciences, Universidade Sagrado Coração, Bauru, São Paulo, Brazil; 3 Department of Biostatistics, Bioscience Institute, Universidade Estadual Paulista, Botucatu, SP, Brazil

**Keywords:** urinary incontinence, pregnancy, diabetes *mellitus*, maternal obesity, incontinencia urinaria, gravidez, diabetes *mellitus*, obesidade materna

## Abstract

**Objective** The lack of data on the impact of hyperglycemia and obesity on the prevalence of pregnancy-specific urinary incontinence (PSUI) led us to conduct a cross-sectional study on the prevalence and characteristics of PSUI using validated questionnaires and clinical data.

**Methods** This cross-sectional study included 539 women with a gestational age of 34 weeks who visited a tertiary university hospital between 2015 and 2018. The main outcome measures were the prevalence of PSUI, the International Consultation on Incontinence Questionnaire Short Form (ICIQ-SF), and the Incontinence Severity Index (ISI) questionnaires. The women were classified into four groups: normoglycemic lean, normoglycemic obese, hyperglycemic lean, and hyperglycemic obese. The differences between groups were tested using descriptive statistics. Associations were estimated using logistic regression analysis and presented as unadjusted and adjusted odds ratios.

**Results** Prevalence rates of PSUI were no different between groups. However, significant difference in hyperglycemic groups worse scores for severe and very severe PSUI. When adjusted data for confound factors was compared with normoglycemic lean group, the hyperglycemic obese group had significantly higher odds for severe and very severe forms of UI using ICIQ-SF (aOR 3.157; 95% CI 1.308 to 7.263) and ISI (aOR 20.324; 95% CI 2.265 to 182.329) questionnaires and highest perceived impact of PSUI (aOR 4.449; 95% CI 1.591 to 12.442).

**Conclusion** Our data indicate that obesity and hyperglycemia during pregnancy significantly increase the odds of severe forms and perceived impact of PSUI. Therefore, further effective preventive and curative treatments are greatly needed.

## Resumo

**Objetivo** A falta de dados sobre o impacto da hiperglicemia e obesidade na prevalência de incontinência urinária específica da gravidez (IAPS) nos levou a realizar um estudo transversal sobre a prevalência e características da IAPS usando questionários validados e dados clínicos.

**Métodos** Este estudo transversal incluiu 539 mulheres com idade gestacional de 34 semanas que visitaram um hospital universitário terciário entre 2015 e 2018. As principais medidas de desfecho foram a prevalência de PSUI, o formulário curto do International Consultation on Incontinence Questionnaire (ICIQ-SF) e os questionários do Incontinence Severity Index (ISI). As mulheres foram classificadas em quatro grupos: magras normoglicêmicas, obesas normoglicêmicas, magras hiperglicêmicas e obesas hiperglicêmicas. As diferenças entre os grupos foram testadas por meio de estatística descritiva. As associações foram estimadas usando análise de regressão logística e apresentadas como odds ratio não ajustadas e ajustadas.

**Resultados** As taxas de prevalência de PSUI não foram diferentes entre os grupos. No entanto, houve diferença significativa nos grupos hiperglicêmicos com piores escores para PSUI grave e muito grave. Quando os dados ajustados para fatores de confusão foram comparados ao grupo magro normoglicêmico, o grupo obeso hiperglicêmico teve chances significativamente maiores de formas graves e muito graves de IU usando ICIQ-SF (aOR 3,157; IC 95% 1,308 a 7,263) e ISI (aOR 20,324; 95% CI 2,265 a 182,329) questionários e maior impacto percebido de PSUI (aOR 4,449; 95% CI 1,591 a 12,442).

**Conclusão** Nossos dados indicam que a obesidade e a hiperglicemia durante a gravidez aumentam significativamente as chances de formas graves e o impacto percebido da PSUI. Portanto, tratamentos preventivos e curativos mais eficazes são extremamente necessários.

## Introduction

Urinary incontinence (UI) may be a very common experience during a woman's lifetime, 1 with a robust influence on well-being and quality of life, as well as an immense economic burden for health services. 2 Estimates of the prevalence and incidence of UI depend on the definitions of the study type and population. Previous epidemiological data showed that the prevalence of UI in women older than 20 years was 23.4–26.4% in the United States. 3 In Brazil, it is considered a common health problem, with an estimated prevalence rate of 27%. 4 Therefore, UI is an important public health concern.

Pregnancy appears to be a major risk factor, particularly during late gestation. 5 In general population, the risk of UI during pregnancy is 18–75%. 6 The term pregnancy-specific UI (PSUI) is used to define any urinary leakage onset during pregnancy. 7 The risk of UI increases as pregnancy progresses due to anatomical and hormonal changes. 6 8 Despite certain risk factors being established for PSUI, some risk factors, such as gestational diabetes mellitus (GDM), are still under consideration. Although some perinatal morbidities related to GDM are associated with UI, GDM alone is considered an independent risk factor for all UI types on post-partum. 9 Taken together, these studies provide compelling evidence for an association between GDM and post-partum UI. Likewise, women with a previous diagnosis of GDM have a well-known increased risk to develop type 2 diabetes mellitus (20–50%) by 10 years postpartum. 10 Obesity (body mass index [BMI] > 30 kg/m ^2^ ) and weight gain during pregnancy are some of the main modifiable risk factors for the development of postpartum diabetes. 11 In the United States, from 1999 to 2010, obesity increased from 28.4% to 34% in women aged 20–39 years. 12 Moreover, 15–20% of mothers have pre-pregnancy obesity 13 and 20–40% experience excessive weight gain during pregnancy. 14 Increased BMI has consistently been reported to play a role in the occurrence of clinical UI. 15

Given that the prevalence of obesity has increased in recent decades, and it is one of the most common medical conditions in women of reproductive age, 16 the premise that obesity and diabetes are linked and are considered a prominent risk factor for developing UI is concerning. Despite compelling epidemiologic data supporting the association of GDM and post-partum UI, 9 as well as obesity and UI, 17 little is known about how hyperglycemia and concurrent obesity might affect the severity of PSUI. Furthermore, current international clinical practice guidelines for UI management fail to present specific recommendations for pregnant women with comorbid conditions, including GDM and obesity, and the treatment of such patients remains a neglected aspect of care. 18 19 Therefore, we hypothesized that GDM and obesity are associated with higher odds of PSUI severity.

The present study aimed to investigate the prevalence of PSUI in a population using questionnaires and clinical data to assess the possible associations between PSUI severity, GDM, and obesity.

## Methods

This cross-sectional study focuses on the relationship between UI, obesity, and GDM. All pregnant women were recruited at the time of prenatal care follow-up at the University Hospital from the Perinatal Diabetes Research Centre (PDRC) of Botucatu Medical School/UNESP/Brazil between 2015 and 2018 and were screened for GDM.

We identified four groups of patients categorized as normoglycemic lean (NL), normoglycemic obese (NO), hyperglycemic lean (HL), and hyperglycemic obese (HO). The diagnosis of GDM was established between the 24th and 28th gestational weeks, using the 75-g oral glucose tolerance test (OGTT) according to the American Diabetes Association criteria 20 and glycemic profile. 21 22 All women with positive screening results for GDM or altered glycemic profiles were classified as hyperglycemic. Glycemic control of women following a diagnosis of hyperglycemia followed the protocol in PDRC. The protocol includes a team of healthcare professionals that encourage adequate nutrition, exercise, and insulin administration. 21 The cut-off for obesity was a BMI of > 30 kg/m ^2^ (calculated using the participant's height and weight). 23 The inclusion criteria were restricted to women with singleton pregnancies who underwent an OGTT between 24 and 28 weeks of pregnancy with a new onset of urinary leakage during pregnancy. Pre-pregnancy UI, known type 1 or type 2 diabetes mellitus, preterm delivery (< 37 weeks of gestation), multiple pregnancies, known fetal anomaly, or any clinical condition that may have jeopardized the health status of the woman were considered as the exclusion criteria.

Data on baseline information (age, parity, pre-pregnancy and current BMI, weight gain during pregnancy, educational level, marital status, fasting glucose, and glycosylated hemoglobin) were collected during the interview at of 34 weeks of gestation and medical records assessment. The Brazilian version of the Incontinence Severity Index (ISI) was used to categorize incontinence severity. 24 The multiplicative score is based on two questions assessing the frequency and volume of incontinence. 25 Women were also asked to complete the Brazilian version of the International Consultation on Incontinence Questionnaire-Urinary Incontinence Short Form (ICIQ-UI SF). 26 The ICIQ-UI SF comprises three scored items and one non-scored item, making it possible to assess the prevalence, severity, interference in daily life, and type of UI. 26 The ICIQ-UI SF score ranges from 0 to 21. Scores on the perceived impact of those reporting UI are set from ‘0’ as not at all to ‘10’ as a great deal. One non-scored item of the ICIQ-UI SF includes eight answers and is a self-diagnostic item to understand the participant's perception of the cause and type of leakage. A form completed immediately after birth was used to record the labor process, mode of delivery, and neonatal birth profile.

The primary outcome was the PSUI prevalence among the groups. UI was classified according to the International Continence Society guidelines for stress UI (SUI) (involuntary leakage on effort or exertion, sneezing, or coughing), urge UI (UUI) (involuntary leakage accompanied by or immediately preceded by urgency), and mixed UI (MUI) (involuntary leakage associated with urgency and exertion, effort, sneezing, or coughing). 27 Secondary outcomes were the prevalence of SUI, UUI, and MUI, as well as the frequency of UI, amount of leakage, the ISI score, the ICIQ-UI score, and perceived impact of UI.

SAS version 9.4 for Windows (Statistical Analysis System Institute Inc., USA) was used for statistical analyses. Clinical features are presented as frequencies and percentages or as means with standard deviations. Differences between groups were tested using chi-square or analysis of variance followed by the Tukey–Kramer analysis. A logistic regression model was used to assess the association between GDM and obesity and UI. Only clinical features with a p-value < 0.05 were included in the adjusted logistic regression analysis (age, gestational age, parity, previous newborn weight, hypertension, newborn weight, and classification).

This study was approved by the Research Ethics Committee of the institution (CAAE: 41570815.0.0000.5411). All patients were informed about the purpose of the study, and those who agreed to participate signed a consent form before recruitment.

## Results

Among the 563 women eligible for recruitment, 539 (95.7%) agreed to participate in the present study. Among these patients, 172 participants were included in the NL group (31.91%), 113 in the NO group (20.97%), 109 in the HL group (20.22%), and 145 in the HO group (26.90%). Baseline characteristics differed between groups, including clinical features such as age, gestational age, parity, previous newborn weight, hypertension, newborn weight, and classification. The background variables of the study population are shown in Table 1 .

**Table 1:** Clinical features of the study population

	Total population ( *n* = 539)	Normoglycemic Lean ( *n* = 172) 31.91%	Normoglycemic Obese ( *n* = 113) 20.97%	Hiperglycemic Lean ( *n* = 109) 20.22%	Hiperglycemic Obese ( *n* = 145) 26.90%	*p* -value between groups
Age (years)	29.12 (6.44)	27.20 (6.15)	28.12 (6.47)	29.73 (7.15) a	31.68 (5.25) a b	
Gestational age (weeks)	36.85 (1.58)	37.01 (1.57)	37.30 (1.63)	36.54 (1.51) a	36.54 (1.52) a b	
Parity	1.11 (1.02)	1.02 (0.99)	0.97 (1.03)	1.06 (0.98)	1.37 (1.04) a b c	
Previous newborn (g)	2237.27 (1601.06)	1950.31 (1590.17)	2221.50 (1590.23)	2188.87 (1655.11)	2627.24 (1518.14) a	
Weight gain during pregnancy (kg)	10.34 (7.59)	13.15 (6.57)	9.16 (9.05) a	11.8 (6.52)b	6.74 (6.57) a b c	
Prepregnancy BMI (kg/m ^2^ )	30.46 (7.46)	24.34 (3.27)	36.44 (5.12) a	25.49 (3.24) b	36.77 (5.93) a c	
Pregnancy BMI (kg/m ^2^ )	34.52 (7.21)	29.53 (3.97)	40.37 (7.01) a	30.18 (4.13) b	39.16 (5.70) a c	
OGTT (mg/dL)						
Fasting	82.86 (16.42)	72.24 (7.65)	76.27 (7.59)	89.69 (17.06) a b	94.74 (17.07) a b c	
1 hour	134.77 (40.78)	107.50 (23.88)	115.58 (26.64)	159.72 (37.04) a b	166.65 (36.28) a b	
2 hours	117.39 (56.78)	96.27 (20.10)	110.63 (98.77) a	136.59 (39.41) a b	135.22 (38.69) a b	
Glycemic mean (mg/dL)	90.31 (13.54)	82.28 (8.46)	84.33 (7.98)	96.24 (10.36) a b	99.43 (15.67) a b	
HbA1c	5.24 (0.55)	4.91 (0.42)	5.12 (0.47) a	5.33 (0.41) a b	5.59 (0.59) a b c	
Hypertension	161 (29.87%)	29 (16.86%)	56 (49.56%)	18 (16.51%)	47 (40%)	<.0001
Race						
White	361 (66.98%)	127 (73.84%)	79 (69.91%)	67 (61.47%)	88 (60.69%)	0.0605
Non-white	178 (33.02%)	45 (26.16%)	34 (30.09%)	42 (38.53%)	57 (39.31%)	
Smoker	53 (9.83%)	17 (9.88%)	9 (7.96%)	13 (11.93%)	14 (9.66%)	0.8038
Vaginal	202 (40.89%)	78 (53.42%)	44 (40.74%)	38 (36.89%)	42 (30.66%)	0.0011
C-section	292 (59.11%)	68 (46.58%)	64 (59.26%)	65 (63.11%)	95 (69.34%)	
Newborn weight (g)	3367.28 (511.25)	3287.11 (493.22)	3350.09 (480.12)	3337.70 (506.14)	3496.01 (535.51)a	
Newborn weight classification						
SGA	31 (6.39%)	16 (11.19%)	5 (4.72%)	7 (6.86%)	3 (2.24%)	0.0058
AGA	403 (83.09%)	114 (79.72%)	94 (88.68%)	87 (85.29%)	108 (80.60%)	
LGA	51 (10.52%)	13 (9.09%)	7 (6.60%)	8 (7.84%)	23 (17.16%)	

Abbreviations: AGA, appropriate for gestational age; BMI, Body Mass Index; HbA1c, Glycated Hemoglobin; LGA, large for gestational age; OGTT, Oral Glucose Tolerance Test; SGA, small for gestational age.

a *p* < 0.05–indicate significant difference compared with normoglycemic lean group (Tukey-Kramer).

b *p* < 0.05 - indicate significant difference compared with normoglycemic obese group (Tukey-Kramer).

c *p* < 0.05 - indicate significant difference compared with hiperglycemic lean group (Tukey-Kramer).

The overall prevalence of PSUI was 70.87% ( *n* = 382), with no difference in the prevalence or type of UI between groups ( Table 2 ). However, the HO group had more frequent ( *p* < 0.0001) and more abundant ( *p* = 0.0009) higher scores for the perceived impact of UI ( *p* < 0.0001), ICIQ-UI SF ( *p* < 0.0001), and ISI ( *p* < 0.0001) questionnaires ( Table 3 ).

**Table 2:** Prevalence of Pregnancy-Specific Urinary Incontinence (PSUI), stress urinary incontinence (SUI), urge urinary incontinence (UUI) and mixed urinary incontinence (MUI)

		Total population ( *n* = 539)	Normoglycemic Lean ( *n* = 172)	Normoglycemic Obese ( *n* = 113)	Hiperglycemic Lean ( *n* = 109)	Hiperglycemic Obese ( *n* = 145)	*p* -value between groups
**PSUI**	Yes	382 (70.87%)	115 (66.86%)	85 (75.22%)	73 (66.97%)	109 (75.17%)	0.2143
	No	157 (29.13%)	57 (33.14%)	28 (24.78%)	36 (33.03%)	36 (24.83%)	
**PSUI (** ***n*** ** = 382)**	UI	1 (0.26%)	0 (0%)	1 (100%)	0 (0%)	0 (0%)	0.1224
	SUI	152 (39.79%)	51 (44.35%)	26 (30.59%)	35 (47.95%)	40 (36.70%)	
	MUI	201 (52.62%)	57 (49.57%)	50 (58.82%)	30 (41.10%)	64 (58.72%)	
	UUI	28 (7.33%)	7 (6.09%)	8 (9.41%)	8 (10.96%)	5 (4.59%)	

PSUI: Pregnancy-Specific Urinary Incontinence; UI: Urinary incontinence; SUI: stress urinary incontinence; UUI: urge urinary incontinence; MUI: mixed urinary incontinence

**Table 3:** Frequency, duration, amount of leakage, scores for the perceived impact of those reporting UI, ICIQ UI-SF and ISI scores

			Total population ( *n* = 539)	Normoglycemic Lean ( *n* = 172) 31.91%	Normoglycemic Obese ( *n* = 113) 20.97%	Hiperglycemic Lean ( *n* = 109) 20.22%	Hiperglycemic Obese ( *n* = 145) 26.90%	*p* -value between groups §
ICIQ UI-SF	Frequency of incontinence episodes	No leakage	157 (29.13%)	57 (33.14%)	28 (24.78%)	36 (33.03%)	36 (24.83%)	<0.0001
		≤Once/week	146 (27.09%)	63 (36.63%)	38 (33.63%)	22 (20.18%)	23 (15.86%)	
		2–3 times/week	83 (15.40%)	25 (14.53%)	12 (10.62%)	20 (18.35%)	26 (17.93%)	
		Once/day	60 (11.13%)	13 (7.56%)	17 (15.04%)	8 (7.34%)	22 (15.17%)	
		>Once/day	78 (14.47%)	11 (6.40%)	12 (10.62%)	22 (20.18%)	33 (22.76%)	
		All the time	15 (2.78%)	3 (1.74%)	6 (5.31%)	1 (0.92%)	5 (3.45%)	
	Amount of leakage	None	157 (29.13%)	57 (33.14%)	28 (24.78%)	36 (33.03%)	36 (24.83%)	0.0009
		Small	217 (40.26%)	78 (45.35%)	56 (49.55%)	41 (37.61%)	42 (28.97%)	
		Moderate	121 (22.45%)	31 (18.02%)	20 (17.70%)	22 (20.18%)	48 (33.10%)	
		Severe	44 (8.16%)	6 (3.49%)	9 (7.96%)	10 (9.17%)	19 (13.10%)	
		Score perceived impact of those reporting UI ( *n* = 382)	4.16 (3.72)	5.12 (3.01)	5.12 (3.17)	5.26 (3.61)	6.69 (3.11)*#‡	
		ICIQ UI-SF score ( *n* = 539)	7.76 (6.41)	6.49 (5.78)	7.47 (5.93)	7.27 (6.51)	9.64 (6.97) abc	
	Perceived impact of those reporting UI ( *n* = 382)	Not at all	39 (10.21%)	11 (9.57%)	9 (10.59%)	13 (17.81%)	6 (5.50%)	<0.0001
		Mildly	76 (19.9%)	26 (22.61%)	21 (24.71%)	13 (17.81%)	16 (14.68%)	
		Moderately	97 (25.39%)	40 (34.78%)	27 (31.76%)	14 (19.18%)	16 (14.68%)	
		Severely	112 (29.32%)	22 (19.13%)	15 (17.65%)	26 (35.62%)	49 (44.95%)	
		To a great extent	58 (15.18%)	16 (13.91%)	13 (15.29%)	7 (9.59%)	22 (20.18%)	
	ICIQ UI-SF ( *n* = 539)	None	157 (29.13%)	57 (33.14%)	28 (24.78%)	36 (33.03%)	36 (24.83%)	<0.0001
		Slight	61 (11.32%)	20 (11.63%)	15 (13.27%)	15 (13.76%)	11 (7.59%)	
		Moderate	167 (30.98%)	65 (37.79%)	45 (39.82%)	27 (24.77%)	30 (20.69%)	
		Severe	132 (24.49%)	26 (15.12%)	19 (16.81%)	30 (27.52%)	57 (39.31%)	
		Very Severe	22 (4.08%)	4 (2.33%)	6 (5.31%)	1 (0.92%)	11 (7.59%)	
	ISI ( *n* = 382)	Slight	123 (32.20%)	53 (46.09%)	33 (38.82%)	22 (30.14%)	15 (13.76%)	<0.0001
		Moderate	135 (35.34%)	42 (36.52%)	33 (38.82%)	25 (34.25%)	35 (32.11%)	
		Severe	86 (22.51%)	15 (13.04%)	11 (12.94%)	19 (26.03%)	41 (37.61%)	
		Very Severe	38 (9.95%)	5 (4.35%)	8 (9.41%)	7 (9.59%)	18 (16.51%)	

Abbreviations: ICIQ-SF, International Consultation on Incontinence Questionnaire-Urinary Incontinence-Short Form; ISI, Incontinence Severity Index; UI, Urinary incontinence.

a *p* < 0.05–indicate significant difference compared with normoglycemic lean group (Tukey-Kramer).

b *p* < 0.05 - indicate significant difference compared with normoglycemic obese group (Tukey-Kramer).

c *p* < 0.05 - indicate significant difference compared with hiperglycemic lean group (Tukey-Kramer).

* *p* < 0.05–indicate significant difference compared with normoglycemic lean group (Poisson).

# *p* < 0.05 - indicate significant difference compared with normoglycemic obese group (Poisson).

‡ *p* < 0.05 - indicate significant difference compared with hiperglycemic lean group (Poisson).

§ Chi-square test.

Table 4 shows the logistic regression analysis with unadjusted and adjusted UI. Surprisingly, when adjusted for age, gestational age, parity, previous newborn weight, hypertension, newborn weight, and classification, the hyperglycemic group had significantly higher odds of UI severity than the other groups in the study. Furthermore, these groups presented a higher perceived impact of UI, ISI, and ICIQ-UI SF severe scores.

**Table 4:** Unadjusted and adjusted odds ratio in the four groups

		Normoglycemic Lean ( *n* = 172)	Normoglycemic Obese ( *n* = 113)	Hiperglycemic Lean ( *n* = 109)	Hiperglycemic Obese ( *n* = 145)	
**Unadjusted**						***p*** **-value between groups**
			**OR with 95% CI**	**OR with 95% CI**	**OR with 95% CI**	
	PSUI	1	1.505 (0.884 - 2.562)	1.005 (0.604 - 1.674)	1.501 (0.917 - 2.456)	0.2164
PSUI	SUI	1	0.563 (0.312 - 1.016)	1.156 (0.642 - 2.082)	0.727 (0.426 - 1.243)	0.1056
	MUI	1	1.496 (0.847 - 2.643)	0.710 (0.393 - 1.284)	1.447 (0.853 - 2.454)	0.0586
	UUI	1	1.624 (0.565 - 4.669)	1.899 (0.658 - 5.481)	0.742 (0.228 - 2.411)	0.3427
Perceived impact of those reporting UI	Not at all	1	0.745 (0.453 - 1.225)	1.249 (0.769 - 2.030)	0.624 (0.389 - 0.999)	0.0419
	Mildly	1	1.123 (0.581 - 2.170)	0.742 (0.353 - 1.557)	0.589 (0.296 - 1.171)	0.2871
	Moderately	1	0.873 (0.481 - 1.585)	0.445 (0.221 - 0.894)	0.323 (0.168 - 0.621)	0.0021
	Severely	1	0.906 (0.438 - 1.872)	2.338 (1.200 - 4.558)	3.452 (1.897 - 6.282)	<0.0001
	To a great extent	1	1.117 (0.506 - 2.467)	0.656 (0.256 - 1.682)	1.565 (0.773 - 3.168)	0.2690
ICIQ UI-SF	None	1	0.665 (0.390 - 1.132)	0.995 (0.597 - 1.657)	0.666 (0.407 - 1.091)	0.0419
	Slight	1	1.163 (0.568 - 2.380)	1.213 (0.596 - 2.484)	0.624 (0.288 - 1.349)	0.3856
	Moderate	1	1.089 (0.670 - 1.772)	0.542 (0.318 - 0.924)	0.429 (0.259 - 0.713)	0.0008
	Severe	1	1.135 (0.595 - 2.165)	2.132 (1.179 - 3.855)	3.637 (2.132 - 6.204)	<0.0001
	Very Severe	1	2.355 (0.649 - 8.540)	0.389 (0.043 - 3.526)	3.448 (1.074 - 11.072)	0.0580
ISI	Slight	1	0.742 (0.420 - 1.313)	0.505 (0.271 - 0.938)	0.187 (0.097 - 0.360)	<0.0001
	Moderate	1	1.103 (0.619 - 1.967)	0.905 (0.490 - 1.674)	0.822 (0.473 - 1.429)	0.7878
	Severe	1	0.991 (0.430 - 2.282)	2.346 (1.104 - 4.983)	4.020 (2.063 - 7.831)	<0.0001
	Very Severe	1	2.285 (0.720 - 7.249)	2.332 (0.711 - 7.647)	4.350 (1.555 - 12.171)	0.0372
adjusted						
	PSUI	1	0.760 (0.297 - 1.949)	2.439 (1.016 - 5.855)	0.631 (0.256 - 1.557)	0.0238
PSUI	SUI	1	0.567 (0.220 - 1.462)	2.012 (0.664 - 6.099)	0.637 (0.261 - 1.551)	0.1220
	MUI	1	1.241 (0.498 - 3.095)	0.490 (0.158 - 1.513)	1.820 (0.766 - 4.328)	0.1138
	UUI	1	2.372 (0.500 - 11.257)	0.927 (0.135 - 6.352)	0.420 (0.063 - 2.784)	0.3238
Perceived impact of those reporting UI	Not at all	1	0.687 (0.281 - 1.680)	2.066 (0.874 - 4.885)	0.511 (0.214 - 1.216)	0.2222
	Mildly	1	2.221 (0.770 - 6.407)	0.822 (0.212 - 3.182)	0.805 (0.270 - 2.400)	0.2564
	Moderately	1	1.156 (0.445 - 3.001)	0.301 (0.087 - 1.039)	0.300 (0.111 - 0.809)	0.0271
	Severely	1	0.468 (0.126 - 1.737)	3.810 (1.134 - 12.801)	4.449 (1.591 - 12.442)	0.0005
	To a great extent	1	0.920 (0.239 - 3.537)	0.962 (0.195 - 4.747)	1.198 (0.361 - 3.977)	0.9752
ICIQ UI-SF	None	1	0.760 (0.297 - 1.949)	2.439 (0.516 - 5.855)	0.631 (0.256 - 1.557)	0.2381
	Slight	1	0.677 (0.181 - 2.539)	0.600 (0.145 - 2.474)	0.631 (0.180 - 2.217)	0.8412
	Moderate	1	2.081 (0.903 - 4.793)	0.204 (0.071 - 0.584)	0.415 (0.178 - 0.964)	0.0001
	Severe	1	0.438 (0.141 - 1.357)	2.244 (0.885 - 5.691)	3.157 (1.308 - 7.623)	0.0012
	Very Severe	1	3.852 (0.357 - 41.511)	3.389 (0.443 - 33.526)	6.496 (0.662 - 63.742)	0.4536
ISI	Slight	1	0.759 (0.297 - 1.939)	0.214 (0.059 - 0.774)	0.194 (0.072 - 0.527)	0.0042
	Moderate	1	1.739 (0.683 - 4.427)	1.106 (0.377 - 3.242)	0.587 (0.234 - 1.472)	0.1660
	Severe	1	0.208 (0.037 - 1.188)	2.297 (0.617 - 8.547)	3.130 (1.070 - 9.153)	0.0059
	Very Severe	1	6.092 (0.603 - 61.538)	11.709 (1.027 - 133.489)	20.324 (2.265 - 182.392)	0.0381

Abbreviations: ICIQ-SF, International Consultation on Incontinence Questionnaire-Urinary Incontinence-Short Form; ISI, Incontinence Severity Index; MUI, mixed urinary incontinence; PSUI, Pregnancy-Specific Urinary Incontinence; SUI, stress urinary incontinence; UI, Urinary incontinence; UUI, urge urinary incontinence.

## Discussion

To the best of our knowledge, this is the first study to assess the influence of obesity and hyperglycemia on the odds of PSUI severity. This cross-sectional study assessed the prevalence, frequency, amount, perceived impact, and severity of PSUI in women as of 34 weeks of gestation. Overall, a high prevalence (70.87%) of PSUI among the 539 participants. We found the highest odds of PSUI severity and the perceived impact of UI in women with hyperglycemia. Even after adjustment for various confounders, including age, gestational age, parity, previous newborn weight, hypertension, newborn weight and classification, women with hyperglycemia without obesity presented the highest odds of PSUI (adjusted odds ratio [aOR]: 2.43; 95% confidence interval [CI]: 1.01–5.85). We observed a substantial increase in the odds of extremely severe PSUI in the HL (aOR: 11.70; 95% CI: 1.02–133.48) and HO groups (aOR: 20.32; 95% CI: 2.26–182.39). Our logistic regression model found that hyperglycemia alone and hyperglycemia linked to obesity were also associated with severe perceived impact of UI in daily life (aOR: 3.81; 95% CI: 1.13–12.80; aOR: 4.44; 95% CI: 1.59–12.44). The persistence, progression and severity of pelvic floor dysfunction can have a significant impact on women's quality of life. 28

With respect to the baseline characteristics of the present study, this cohort represented the underlying population characteristics of women with hyperglycemia during pregnancy. Advancing maternal age has been recognized as a major risk factor for the development of hyperglycemia during pregnancy. 29 The other risk factors greater parity, increased BMI, and hypertension. 30 31 Our data indicate these risk factors in the present cohort of the hyperglycemic groups. Such risk factors are also associated with an increased risk of developing UI. 6 32 In our study, although women in the HO group presented lower weight gain during pregnancy, which may be related to the fact that they received the treatment at PDRC, the symptoms related to UI appeared to be more severe than those in the other groups.

According to Daly et al., 33 21.7% of the population studied presented women with new-onset leakage who were continent in the 12 months before pregnancy. Brown et al., 34 found that the most common PSUI is SUI, characterized by unintentional loss of urine during physical movement or activity (e.g., sneezing, coughing, running, or heavy lifting). The pathophysiology of PSUI is multifactorial and yet to be understood. It has been implicated that hormonal and mechanical changes may play an important role. 35 In our sample, there was no difference in the prevalence of the UI types between the groups. Studies showed that irrespective of the type, UI has detrimental effects on the quality of life in ∼54.3% of all pregnant women 36 and the quality of life of pregnant women with incontinence worsens with increasing gestational age to term. 37 Our sample presented higher prevalence of PSUI rates (70.87%) when compared the general literature. However, this corresponds with a similar study with smaller sample size, in the same gestational period (i.e., 34–38 weeks of gestation) the prevalence rate was 60.5%. 38 Further research is needed to explore the differences in prevalence of PSUI in multicentric and multi-ethnic groups.

Our findings show that women with a BMI of ≥ 30 kg/m ^2^ are significantly more likely to report less frequent incontinence episodes and amount of leakage, moderately perceived impact of UI, and slight to moderate UI severity. A large longitudinal study that enrolled 10,098 women who were followed up as of 28 weeks of gestation found that high prenatal BMI increased the risk of SUI in late pregnancy (OR: 1.037; 95% CI: 1.020–1.054). 39 Overweight and obesity are considered major modifiable risk factors for UI in young and middle-aged women. 40 Previous studies have shown that middle-aged women with obesity are 3.1 times more likely to have severe UI than women with BMI in the normal range. 41 These differences might be related to the different types of inquiries used to address UI symptoms and study designs. Anatomical changes in patients with obesity assessed by ultrasonography showed that bladder neck descent was more evident in women with obesity than in women with normal weight. 42 A high BMI increases intra-abdominal pressure, resulting in an imbalance between vesical pressure and urethral closure, triggering urine leakage. 15 43

The first study to report the prevalence of UI in women with GDM was conducted by Kim et al. 44 They recruited 228 women with GDM; 49% reported weekly or more episodes of incontinence during pregnancy and 50% after delivery. 44 Another cross-sectional study found that GDM was an independent risk factor (OR: 2.26; 95% CI: 1.116–4.579) for PSUI, and PSUI was a risk factor 2 years post cesarean section UI (OR: 4.992; 95% CI: 1.383–18.023). 45 A large study 9 recruited 6653 women who were followed up for 2 years postpartum to investigate the association between GDM and postpartum UI. They demonstrated that women with GDM were more likely to report SUI (OR: 1.97; 95% CI: 1.56–2.51), UUI (OR: 3.11; 95% CI: 2.18–4.43), and MUI (OR, 2.73; 95% CI: 1.70–4.40). 9 Furthermore, another study showed that the occurrence of PSUI, the severity of UI, and the negative impact of UI on the quality of life are increased in women with hyperglycemia during pregnancy. 38 Recent studies 46 47 conducted in animal models and pregnant women have aimed to identify and quantify the morphological changes in the rectus abdominis muscles due to hyperglycemia during pregnancy. Changes in the fiber type, fiber area, and collagen content have been reported and may be related to diabetic myopathy.

The strengths of this study include the use of validated questionnaires that enable the identification of the type, frequency, severity, and perceived impact of UI. The International Consultation on Incontinence recognized that ICIQ questionnaires are grade A (high-quality) measurement instruments for assessing UI. 48 Another strength of our study is the use of a database with the glycemic values of the participants and the established diagnostic criteria for GDM and obesity. An important limitation is the limited number of participants that could have powered our results and the lack of an objective measure of UI assessment, such as bladder diaries, pad test, and/or urodynamic test, to compare with our subjective measures.

## Conclusion

The results of the present study show that hyperglycemia during pregnancy is an independent risk factor for PSUI. The logistic regression models showed that when compared with the normoglycemic lean women, women who are obese and have hyperglycemia during pregnancy are more likely to experience severe and very severe PSUI with important perceived impact on daily life. The findings from our study provide information on PSUI in volunteers at the third trimester of pregnancy screened for hyperglycemia, and such findings are directly relevant to clinical practice. Such risk factors are preventable, manageable, and even curable, and healthcare professionals should perform evidence-based treatment.
